# AMPK Activator O304 Protects Against Kidney Aging Through Promoting Energy Metabolism and Autophagy

**DOI:** 10.3389/fphar.2022.836496

**Published:** 2022-03-02

**Authors:** Mingsheng Zhu, Weiwei Shen, Jiemei Li, Nan Jia, Yabing Xiong, Jinhua Miao, Chao Xie, Qiyan Chen, Kunyu Shen, Ping Meng, Xiaolong Li, Qinyu Wu, Shan Zhou, Maosheng Wang, Yaozhong Kong, Lili Zhou

**Affiliations:** ^1^ State Key Laboratory of Organ Failure Research, National Clinical Research Center of Kidney Disease, Guangdong Provincial Clinical Research Center for Kidney Disease, Guangdong Provincial Key Laboratory of Nephrology, Division of Nephrology, Nanfang Hospital, Southern Medical University, Guangzhou, China; ^2^ Department of Nephrology, The People’s Hospital of Gaozhou, Maoming, China; ^3^ Department of Nephrology, The First People’s Hospital of Foshan, Foshan, China; ^4^ Department of Central Laboratory, Huadu District People’s Hospital, Southern Medical University, Guangzhou, China; ^5^ The Cardiovascular Center, The People’s Hospital of Gaozhou, Maoming, China

**Keywords:** O304, kidney aging, AMPK, autophagy, energy metabolism

## Abstract

Aging is an important risk factor for kidney injury. Energy homeostasis plays a key role in retarding aging, and mitochondria are responsible for energy production. In the kidney, renal tubular cells possess high abundance of mitochondria to meet the high energy consumption. AMPK is an evolutionarily conserved serine/threonine kinase which plays a central role in maintaining energy homeostasis and mitochondrial homeostasis. Besides that, AMPK also commands autophagy, a clearing and recycling process to maintain cellular homeostasis. However, the effect of AMPK activators on kidney aging has not been fully elucidated. To this end, we testified the effects of O304, a novel direct AMPK activator, in naturally aging mice model and D-Galactose (D-Gal)-treated renal tubular cell culture. We identified that O304 beneficially protects against cellular senescence and aged-related fibrosis in kidneys. Also, O304 restored energy metabolism, promoted autophagy and preserved mitochondrial homeostasis. Transcriptomic sequencing also proved that O304 induced fatty acid metabolism, mitochondrial biogenesis and ATP process, and downregulated cell aging, DNA damage response and collagen organization. All these results suggest that O304 has a strong potential to retard aged kidney injury through regulating AMPK-induced multiple pathways. Our results provide an important therapeutic approach to delay kidney aging.

## Introduction

Aging is one of the most important risk factors for many organ disorders (St et al., 2015; Godic, 2019). It is a process characterized with stem cell depletion, reduced autophagy, mitochondrial dysfunction, impaired immune system, epigenetic changes, somatic and mitochondrial DNA mutations, loss of telomeres, and so on (Jang et al., 2018; Johnson and Stolzing, 2019; Luo and Qin, 2019; Sameri et al., 2020). Organ aging is often accompanied by abnormal energy metabolism. For example, studies have shown that elderly mice have lower energy production which is highly related with glucose intolerance, reduced mitochondrial biogenesis and oxidative phosphorylation, and reduced fatty acid oxidation (Azzu and Valencak, 2017). Inside these factors, lipid metabolism plays an important role in aging process (Johnson and Stolzing, 2019). There are increasing evidences showing that aging is associated with increased lipid accumulation in important organs, especially kidneys (Papsdorf and Brunet, 2019). Reports also show that fatty acid oxidation deficiency in kidneys is an important inducer of renal fibrosis (Chung et al., 2018).

Mitochondria are the most important organelle to be involved in aging process (Chapman et al., 2019). The excessive production of reactive oxygen species (ROS) from mitochondria and accumulation of damaged mitochondria have been proposed as a hallmark of aging (Picca et al., 2019; Wei and Szeto, 2019; Xiong and Zhou, 2019). Kidney is one of the most important organs to keep metabolic stability and maintain the balance of water and electrolyte (Isaka et al., 2021; Scholz et al., 2021; Zhou et al., 2021). In the kidney, renal tubular cells possess high abundance of mitochondria because of their high energy consumption (Higgins and Coughlan, 2014). Mitochondrial dysfunction leads to oxidative stress and inflammation, which further induces cellular senescence in the kidney and accelerates renal fibrosis (Zhou et al., 2012; Su et al., 2019). Hence, mitochondrial homeostasis plays a key role in maintaining normal kidney function. However, the therapeutic strategies have not been investigated in detail.

5ʹ-adenosine monophosphate (AMP)-activated protein kinase (AMPK) is an evolutionarily conserved serine/threonine kinase which plays an important role in maintaining cellular homeostasis, especially energy stabilization (Jeon, 2016), and regulating many other cellular processes (Zhou et al., 2001; Hardie, 2003; Shibata et al., 2004). AMPK is a heterotrimeric complex containing one catalytic α-subunit and two regulatory β- and γ-subunits (Davies et al., 1994). It would be activated by phosphorylation at T172 of the α-subunit by AMP or adenosine diphosphate (ADP) binding to γ-subunit (Hardie et al., 2012). Under full energy conditions, the ratios of AMP/ATP and ADP/ATP are low, resulting that the phosphatases can easily access T172 of the AMPK α-subunit and make it unphosphorylated (Jeon, 2016). On the contrary, AMPK would be phosphorylated in case of energy deficiency and activated by a high AMP/ATP ratio through upstream kinases such as Liver kinase B1 (LKB1). AMPK activation plays a key role in some age-related diseases. For example, AMPK activation could improve cognitive decline through reducing oxidation and inflammation, and stimulating mitochondrial biogenesis (Martin-Montalvo et al., 2013; Ng et al., 2014; Xin and Lu, 2020). Recently, the drug O304 which is an AMPK activator has drawn attention. O304 is a pan-AMPK activator, which increased AMPK activity by suppressing the dephosphorylation of the Thr172 in the α-subunit of AMPK (Steneberg et al., 2018; Das et al., 2019). Studies have shown its blocking effect of O304 on chronic disease or ageing. For example, O304 could reduce fasting plasma glucose levels and insulin resistance, and was able to improve peripheral microvascular perfusion and reduce blood pressure both in animals and T2D patients (Steneberg et al., 2018). Besides, O304 could improve cardiac function and exercise capacity in aged mice, which presents the great potential to improve quality of life in aged individuals (Ericsson et al., 2021).

AMPK is highly involved in autophagy, a process that regulating wastes recycling (Salminen and Kaarniranta, 2012). Autophagy is a lysosomal degradation pathway in which cellular components are degraded and recycled to maintain cellular homeostasis (Ma et al., 2018), and autophagy deficiency is highly related with ageing and organ dysfunction (Garcia-Prat et al., 2016). For example, inhibiting mammalian target of rapamycin (mTOR) could increase autophagy and further lead to lifespan extending (Harrison et al., 2009) or alleviate age-related organ dysfunction (Caccamo et al., 2010). In the kidney, autophagy is also crucial for physiologic and pathologic response of kidney cells (Tang et al., 2020). Notably, autophagy is regulated by AMPK signaling. Studies show that autophagy is promoted by AMPK by phosphorylating autophagy-related protein mTORC1 or indirectly by regulating autophagy-related genes FOXO3, TFEB, and BRD4 (Kim et al., 2011; Li and Chen, 2019). However, the role of AMPK activation in autophagic pathways in kidney aging has not been fully elucidated.

In this study, we testified the therapeutic effects of O304, a novel AMPK activator, on kidney aging in naturally aging mice model and D-Gal-cultured human proximal renal tubular cells. The results show that O304 can retard kidney aging through AMPK-induced fatty acid metabolism and autophagy. All these results suggest that O304 may represent a novel therapeutic potential for age-related kidney injury.

## Material and Methods

### Animal Models

Male C57BL/6 mice (2-, 18- and 24-month-old mice) were purchased from the Experimental Animal Center of Southern Medical University (Guangzhou, China). All experiments involving animals were approved by the Animal Ethics Committee at Southern Medical University. To testify the effects of O304 on the naturally aging mice, unilateral nephrectomy was firstly performed in the 18-month-old mice. One week after the surgery, some mice were sacrificed as control group, while other mice were injected intraperitoneally with saline solution or O304 (HY-112233; MedChemExpress) (10 mg/kg/d) every day for 12 weeks. The detailed experimental designs were shown in Figure 3A. Then all the mice were sacrificed and tissues were collected for various analyses.

### Cell Culture and Treatment

Human proximal tubular epithelial cells (HKC-8) were provided by Dr. L. Racusen (Johns Hopkins University, Baltimore, MD, United States) and cultured in DMEM/F12 medium (Biological Industries) supplemented with 10% fetal bovine serum (Biological Industries). HKC-8 cells were pretreated with O304 at 50 nM for 1 h, followed by treatment with D-Gal (G0750; Sigma-Aldrich) at 10 mg/ml for 60 h. Then the whole-cell lysates were prepared and subjected to western blot analyses. Besides, HKC-8 cells cultured on coverslips were also detected by immunofluorescence.

### Western Blot Analysis

Protein expression was analyzed by western blot analysis. Briefly, total proteins were extracted from cells or renal tissues with lysis buffer and the protein concentrations were measured using BCA protein concentration determination. Then total proteins were quantified and subjected to SDS-PAGE electrophoresis. After that, the proteins were transferred to a PVDF (polyvinylidene fluoride) membrane (Merck Millipore), blocked in 1% bovine serum albumin for 1 h and incubated with primary antibodies at 4°C overnight. The next day, the PVDF membrane were incubated with a secondary horseradish peroxidase-conjugated antibody for 1 h at room temperature. The antigen-antibody complexes were visualized by the ECL kit (Applygen, Beijing, China). The primary antibodies included: anti-fibronectin (F3648; Sigma-Aldrich), anti-PGC-1α (ab54481; Abcam), anti-p-AMPK (2535s; Cell Signaling Technology), anti-AMPK (2532s; Cell Signaling Technology), anti-p-mTOR (ab109268; Abcam), anti-mTOR (ab32028; Abcam), anti-P62 (ab109012; Abcam), anti-LC3B (ab48394; Abcam), anti-α-SMA (a2547; Sigma-Aldrich), anti-α-SMA (ab5694; Abcam), anti-TFAM (PB0413; Boster), anti-P16^INK4A^ (ab189034; Abcam), anti-P19^ARF^ (ab26696; Abcam), anti-γH_2_AX (ab26350; Abcam), anti-TOMM20 (ab186735; Abcam), anti-CPT1A (ab128568; Abcam), anti-ACOX1 (A8091; ABclonai), anti-active β-catenin (19807s, Cell Signaling Technology), anti-β-actin (RM 2001; Ray Antibody Biotech) and anti-α-tubulin (RM 2007; Ray Antibody Biotech).

### Immunofluorescence Staining

HKC-8 cells cultured on coverslips or kidney cryosections (3 µm) were fixed with 4% paraformaldehyde for 15 min at room temperature, followed by permeabilizing with 0.2% of Triton X-100 (T8787; Sigma-Aldrich) for 10 min and blocking with 10% of donkey serum for 1 h. Then the slides were immunostained with special primary antibodies overnight at 4°C. The primary antibodies included: anti-fibronectin (F3648; Sigma-Aldrich), anti-γH_2_AX (ab26350; Abcam), anti-TOMM20 (ab186735; Abcam), anti-mTOR (ab32028; Abcam), anti-P62 (ab207305; Abcam), anti-ADFP (ADRP) (ab52356; Abcam), anti-active β-catenin (19807s, Cell Signaling Technology) and anti-LC3B (ab48394; Abcam). The next day, the slides were stained with a Cy3-or Cy2-conjugated secondary antibody (Jackson ImmunoResearch Laboratories) for 1 h, followed by DAPI (Sigma-Aldrich) staining for 10 min. Finally, Images were taken by confocal microscopy (Leica TCS SP2 AOBS, Leica Microsystems, Buffalo Grove, IL).

### Detection of Autophagic Flux

Cells were cultured on coverslips and incubated with lentiviruses expressing RFP-GFP-LC3B (HANBIO, Shanghai, China), after which they were treated in different groups as indicated. The RFP-GFP-LC3B sensor enables detection of neutral-pH LC3B-positive autophagosomes (green fluorescence) and acid-pH LC3B-positive autophagolysosomes (red fluorescence). Once an autophagosome fuses with a lysosome, the pH would become acid, leading to quenching of the GFP signal (whereas RFP is unaffected).

### Immunohistochemical Staining and Transmission Electron Microscopy

For immunohistochemical staining, 3-µM-thick paraffin sections of kidney samples were prepared. Immunohistochemical staining was performed using routine protocol. After incubation with primary antibodies, the antigen/antibody complexes were detected with biotinylated secondary antibody (Jackson ImmunoResearch), amplified with ABC Elite peroxidase (Vector Laboratories), and detected by AEC (Vector Laboratories). Images were taken by a microscope DP 27 CCD camera (Olympus, Japan). The primary antibodies used as follows: anti-p-AMPK (2535s; Cell Signaling Technology), anti-p-mTOR (ab109268; Abcam), anti-CPT1A (ab128568; Abcam) and anti-fibronectin (F3648; Sigma-Aldrich). For transmission electron microscopy (TEM), kidney cortex and HKC-8 cells cultured on coverslips were collected and fixed in 1.25% glutaraldehyde/0.1 M phosphate buffer before detection. Ultrathin sections (60 nm) were prepared by a routine procedure and examined under an electron microscope (JEOL JEM-1010).

### SA-β-Gal Staining

3-µM-thick frozen sections of kidney tissue were used to assess renal senescence by the detection of β-galactosidase activity (#9860; Cell Signaling Technology) according to the manufacturer’s instructions. Briefly, frozen sections were washed with PBS and fixed for 15 min at room temperature, then incubated in the SA-β-gal staining solution overnight at 37°C. Pictures were taken under the microscope (JEOL JEM-1010).

### Reverse Transcription and Real-Time PCR

Total RNA was extracted using TRIzol RNA isolation kits (Life Technologies, Grand Island, NY) according to manufacturer’s instructions. cDNA was synthesized using 1 μg of RNA in 20 μL of reaction buffer containing qRT SuperMix (R323-01; Vazyme) and random primers. Real-time PCR was carried out with a qPCR SuperMix kit (Q341-02; Vazyme) according to manufacturer’s instructions. The sequences of the primers were as following: mouse PPARα, 5′-GGA​ACC​CAA​GTT​TGA​CTT​CGC-3′ and 5′-CCC​CTC​CTG​CAA​CTT​CTC​AAT-3’; mouse CPT1A, 5′-CAA​GGT​CTT​CTC​GGG​TCG​AAA-3′ and 5′-CAC​TGC​AAT​TTG​GGT​CCA​AGG-3’; mouse CPT2, 5′-TCC​GCT​TTG​TTC​CTT​CCT​CTC-3′ and 5′-CAT​CAC​GAC​TGG​GTT​TGG​GTA-3’; mouse ACOX1, 5′-AAC​TTC​CTC​ACT​CGA​AGC​CAG-3′ and 5′-AAG​GTC​CAA​AGG​CTC​AGG​ATG-3’; human PPARα, 5′-AAC​GAT​TCG​ACT​CAA​GCT​GGT-3′ and 5′-TGT​GAC​ATC​CCG​ACA​GAA​AGG-3’; human ACOX1, 5′-AAC​CCG​GAG​CTG​CTT​ACA​C-3′ and 5′-GGC​TGC​GAG​TGA​GGA​AGT​T-3’.

### mitoSOX Staining

3-µM-thick frozen sections of kidney tissue or HKC-8 cells cultured on coverslips were used to detect renal mitochondrial ROS production *via* mitoSOX staining (M36008; Thermo Fisher) according to the manufacturer’s instructions. For kidney tissues, frozen sections were fixed with 4% paraformaldehyde for 15 min at room temperature and then incubated in the mitoSOX solution for 10 min. For HKC-8 cells, cells cultured on coverslips in 6-well plates were co-treated with O304 and D-Gal for 60 h. After that, mitoSOX solution was added to each well in the concentration of 5 μM. After mitoSOX incubation for 2 h in 37°C, the cells were fixed with 4% paraformaldehyde for 15 min at room temperature. After counterstaining with DAPI, images were taken by confocal microscopy (Leica TCS SP2 AOBS, Leica Microsystems, Buffalo Grove, IL) or Olympus DP80 microscope with EMCCD camera.

### Transcriptomic Analysis

Total RNA was extracted using RNA isolation kits according to manufacturer’s instructions. The steps of transcriptome sequencing analysis include RNA quantification and qualification, library preparation, and clustering and sequencing. After quality control, clean data (clean reads) were obtained by removing low-quality reads and the reads containing adapter or N base from raw data. Reference genome and gene model annotation files were downloaded from genome website directly. Index of the reference genome was built and paired-end clean reads were aligned to the reference genome using HISAT2 v2.0.5. FeatureCounts v1.5.0-p3 was used to count the reads numbers mapped to each gene. And then FPKM of each gene was calculated based on the length of the gene and reads count mapped to this gene. Differential expression analysis of different groups was performed using the DESeq2 R package (1.20. 0). Gene Ontology enrichment analysis of differentially expressed genes was implemented using the clusterProfiler R package, in which gene length bias was corrected. Besides, clusterProfiler R package was also used to test the statistical enrichment of differential expression genes in Kyoto Encyclopedia of Genes and Genomes (KEGG) database pathways. Gene Set Enrichment Analysis (GSEA) was performed using GSEA web interface with the Molecular Signature Database“Reactome mitochondrial biogenesis,” “GOBP aging,” “GOBP fibroblast activation,” “GOBP fibroblast proliferation,” “GOBP epithelial differentiation,” “Reactome mitochondrial fatty acid beta oxidation,” “GOBP reactive oxygen species metabolic process” and “GOBP autophagy of mitochondrion” genesets, to reveal the significant consistent difference between two groups. *p* < 0.05 was considered to be statistically significant. The heatmap were generated using Hiplot (https://hiplot.com.cn), a comprehensive web platform for scientific data visualization. The transcriptome sequencing analysis of the research is supported by Novogene Co., Ltd. (Beijing, China).

### Nile Red Staining

HKC-8 cells cultured on coverslips were fixed with 4% paraformaldehyde for 10 min at room temperature, followed by permeabilization with 0.1% Tween 20 for 5 min. Then the cells were incubated with a Nile red (Sigma 72458) and DAPI (Sigma-Aldrich) dual staining solution in the dark room for 10 min. Images were taken by confocal microscopy (Leica TCS SP2 AOBS, Leica Microsystems, Buffalo Grove, IL).

### Statistical Analyses

Statistical analysis was carried out by SPSS 20.0 (SPSS Inc., Chicago, IL). Data were reported as the mean ± the standard error of the mean. All data were firstly analyzed for normal distribution using the D’Agostino normality test before comparison. Statistical significance was evaluated by either one-way ANOVA or Student’s t-test. For comparison of two groups, comparisons were made by t’-test, while one-way ANOVA was used for analyzing the difference among multiple groups. A value of *p* < 0.05 was considered to be statistically significant.

## Results

### Aged Kidney is Associated with Downregulation of AMPK and Dysfunction of Fatty Acid Metabolism and Autophagy

**FIGURE 1:**
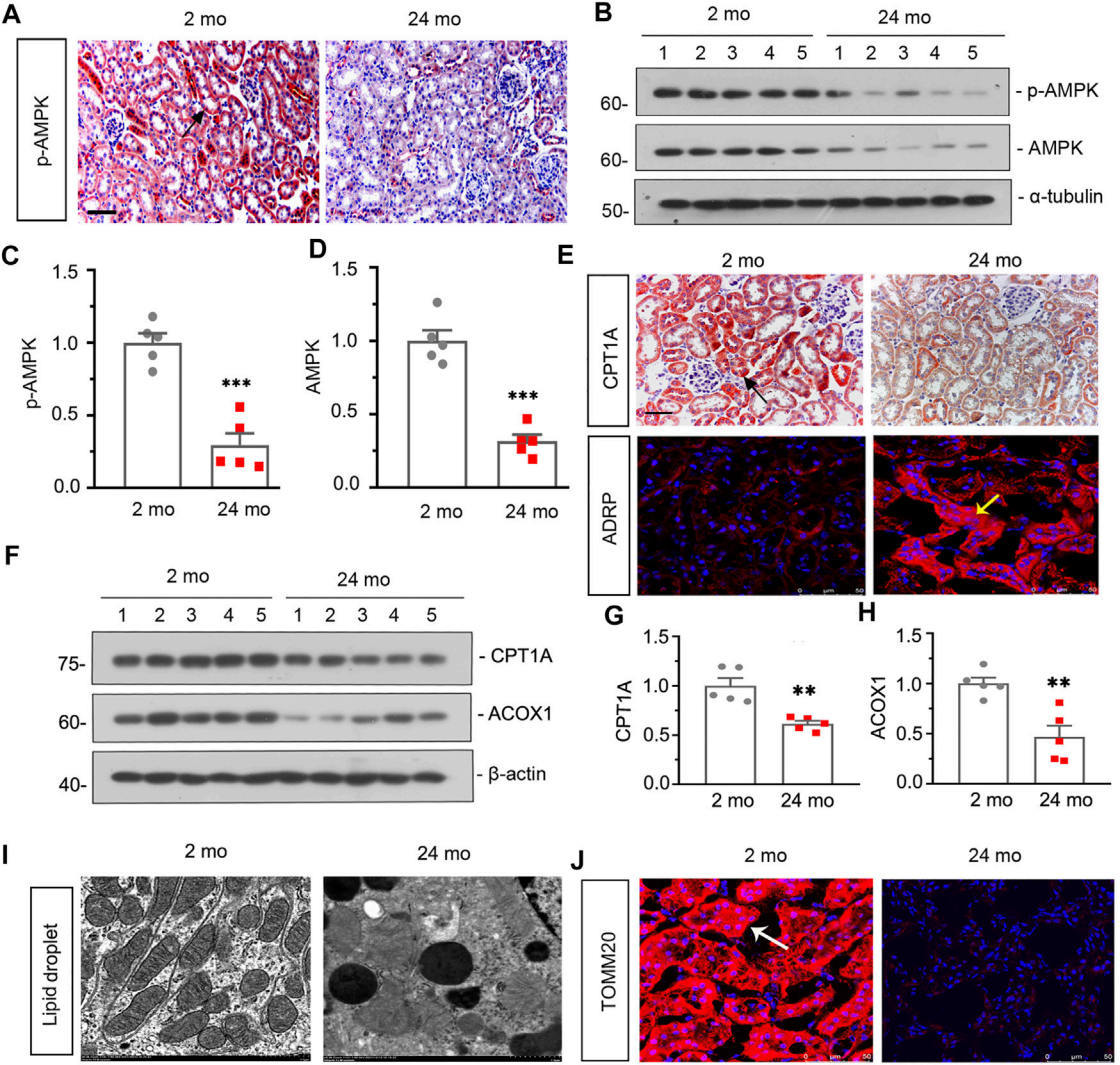
Aged kidney is associated downregulation of AMPK and dysfunction of fatty acid metabolism and mitochondria **(A)** Representative micrographs showing p-AMPK expression in kidneys in 2-month-old and 24-month-old mice. Paraffin-embedded kidney sections were immunostained with an antibody against p-AMPK. Arrows indicate positive staining. scale bar, 50 μm. **(B–D)** Representative western blot and quantitative data showing renal expression of p-AMPK and AMPK in 2-month-old and 24-month-old mice. Numbers (1–5) indicate each individual animal in each given group. ***p < 0.001 versus 2-month-old mice group. **(E)** Representative micrographs showing CPT1A (top) and ADRP (bottom) expression in kidneys in two groups. Paraffin-embedded kidney sections were immunostained with an antibody against CPT1A. Cryosections were subjected to fluorescence staining for ADRP. Arrows indicate positive staining. scale bar, 50 μm. **(F–H)** Representative western blot and quantitative data showing renal expression of CPT1A and ACOX1 in 2-month-old and 24-month-old mice. Numbers (1–5) indicate each individual animal in each given group. **p < 0.01 versus 2-month-old mice group. **(I)** Representative transmission electron microscopy (TEM) micrographs show fat droplet in ultrathin kidney sections. **(J)** Representative micrographs showing TOMM20 expression in kidneys in 2-month-old and 24-month-old mice. Cryosections were subjected to fluorescence staining for TOMM20. Arrows indicate positive staining.

Firstly, we assessed the expression of p-AMPK in the kidneys in 2- and 24-month-old mice. As shown in Figure 1A, compared with 2-month-old mice, immunohistochemical staining of kidneys showed that the expression level of p-AMPK was down-regulated in 24-month-old mice. Besides, similar result was demonstrated by analysis of p-AMPK and AMPK *via* western blot (Figures 1B–D). We next assessed the effects of aging on the fatty acid metabolism. As shown in Figure 1E, the expression of carnitine palmitoyl transferase-1A (CPT1A) detected by immunohistochemical staining was greatly down-regulated in 24-month-old mice, while the expression of adipose differentiation related protein (ADRP) detected by immunofluorescent staining was up-regulated. Besides, western blot analyses showed that the expression of CPT1A and Acyl-CoA oxidase-1 (ACOX1) was also greatly down-regulated in aged mice (Figures 1F–H). What’s more, fat droplet could be easily found in aged mice by transmission electron microscopy (Figure 1I). These results confirmed that aged kidney is associated with dysfunction of fatty acid metabolism.

**FIGURE 2:**
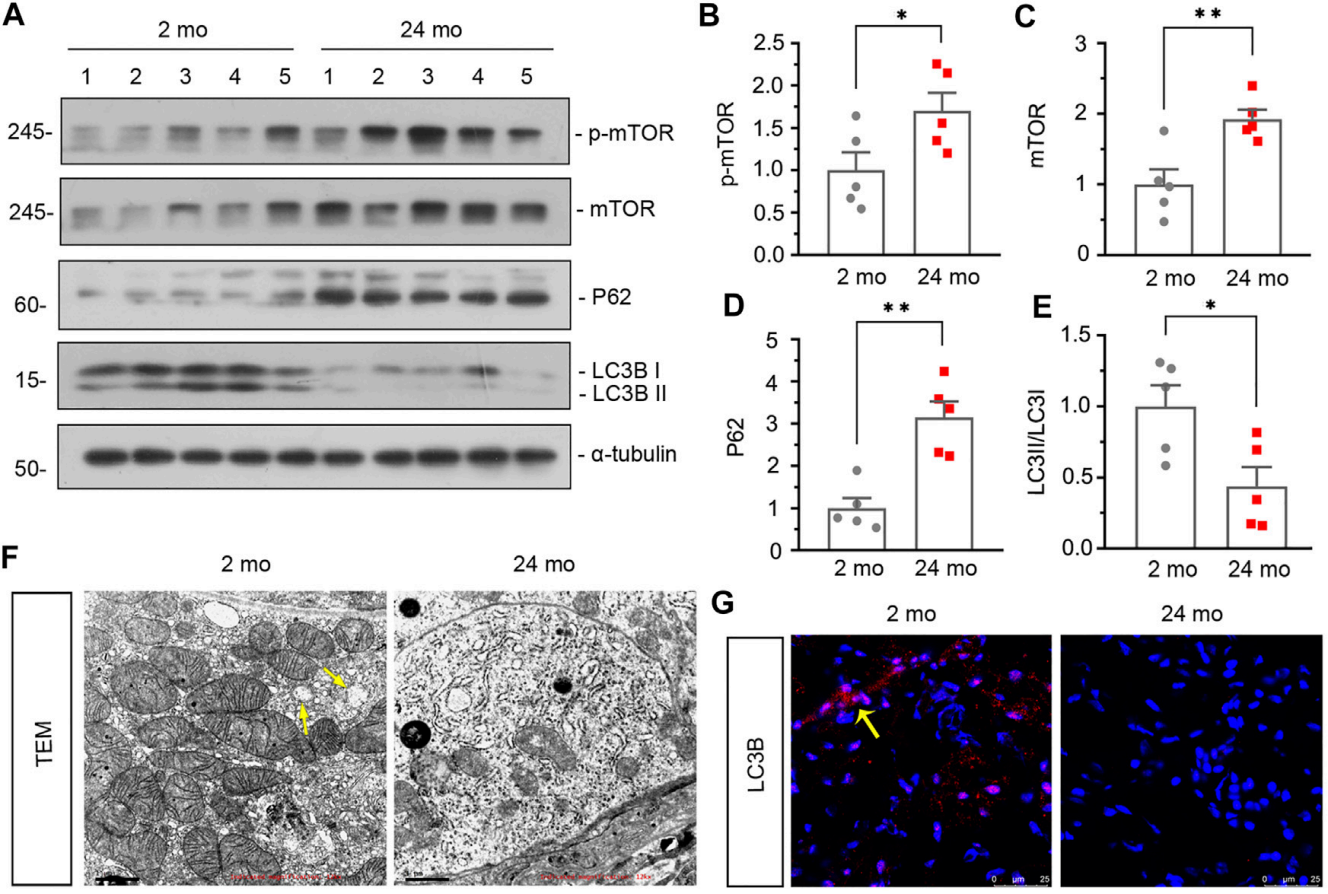
Aged kidney is associated dysfunction of autophagy. **(A–E)** Representative western blot and quantitative data showing renal expression of p-mTOR, mTOR, P62 and enhanced LC3-II formation in 2-month-old and 24-month-old mice. Numbers (1–5) indicate each individual animal in each given group. *p < 0.05, **p < 0.01 versus 2-month-old mice group. **(F)** Representative transmission electron microscopy (TEM) micrographs showing mitochondrial ultrastructure morphology and autophagic vacuole in ultrathin kidney sections. Arrow indicates autophagic vacuole. Scale bar, 1 μm. **(G)** Representative micrographs showing LC3B expression in kidneys in 2-month-old and 24-month-old mice. Cryosections were subjected to fluorescence staining for LC3B. Arrows indicate positive staining.

Then we assessed the effect of aging on renal autophagy. As shown in Figure 1J, compared with 2-month-old mice, the mitochondrial function was impaired in aged mice since the expression of translocase of outer mitochondrial membrane 20 (TOMM20) was down-regulated. In addition, western blot analyses showed that the expressions of p-mTOR, mTOR and P62 (Figures 2A–D) were also greatly increased in aged mice, while the conversion of LC3-I to LC3-II (Figures 2A,E) was down-regulated. Besides, similar result was demonstrated by immunofluorescent staining for LC3B (Figure 2G). Furthermore, transmission electron microscopy was used to detect autophagic vacuole and the ultrastructure of mitochondria and the results showed that autophagic vacuole and intact mitochondria were found in 2-month-old mice rather than aged mice (Figure 2F). These results confirmed that aged kidney is associated with autophagy dysfunction.

### O304 Could Activate AMPK, Alleviate Renal Senescence and Fibrosis, and inhibit β-catenin in Naturally Aging Mice

**FIGURE 3:**
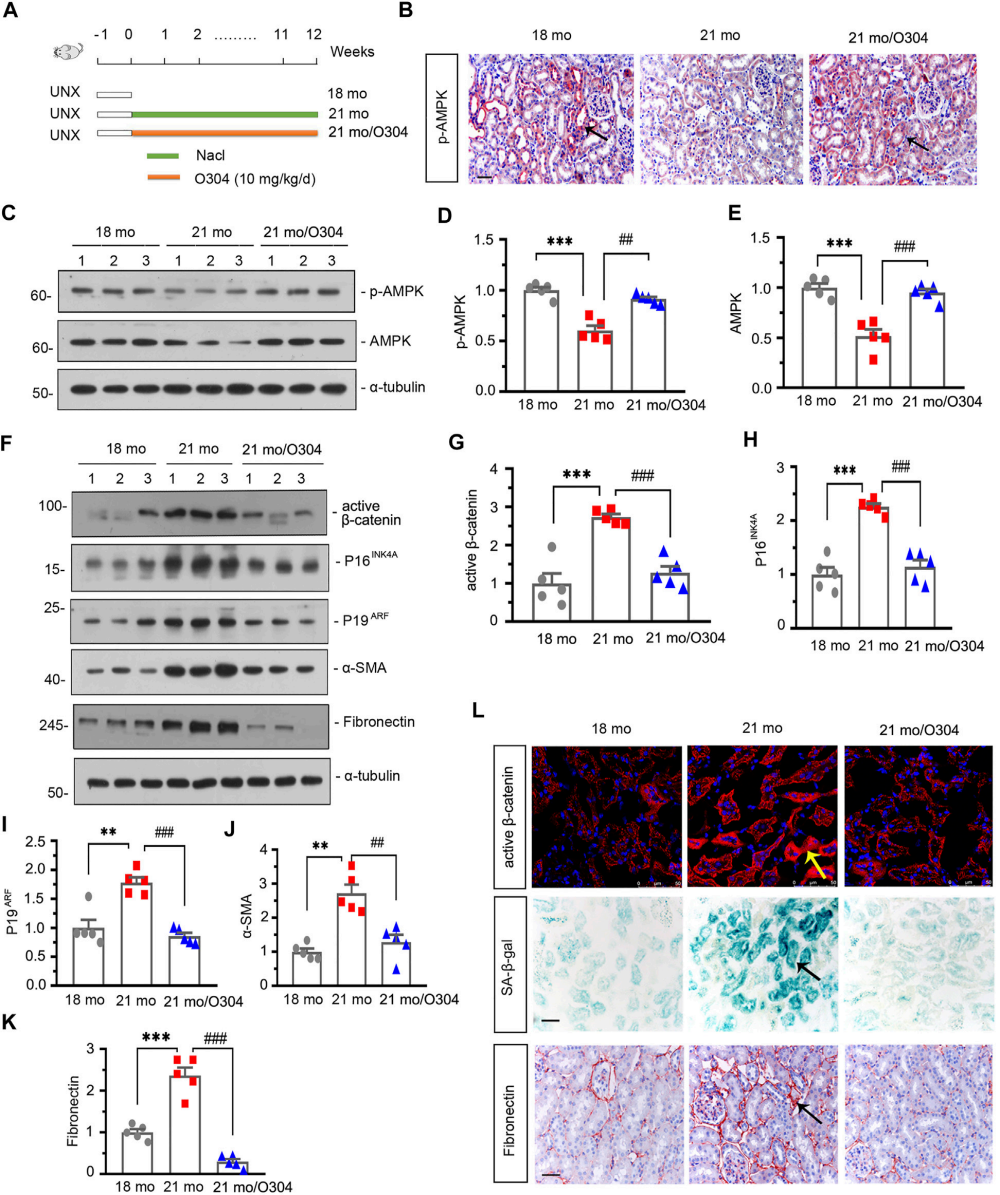
O304 could activate AMPK, alleviate renal senescence and fibrosis, and inhibit β catenin in naturally aging mice. **(A)** Experimental design. White bar indicated the time after the surgery was 1 week. Green bar indicated mice were administered intraperitoneal injections of 0.9% sodium chloride solution for 12 weeks after surgery. Orange bar indicated mice were administered intraperitoneal injections of O304 at 10 mg/kg/day for 12 weeks after surgery. UNX: unilateral nephrectomy. **(B)** Representative micrographs showing renal expression of p-AMPK in different groups. Paraffin-embedded kidney sections were immunostained with antibody against p-AMPK. Arrows indicate positive staining. Scale bar, 50 μm. **(C–E)** Representative western blot and quantitative data showing renal expression of p-AMPK and AMPK in different groups. Numbers (1–3) indicate each individual animal in each given group. ***p < 0.001 versus 18-month-old mice group. ^##^ *p* < 0.01, ^###^ *p* < 0.001versus 21-month-old mice group alone (*n* = 5). **(F–K)** Representative western blot and quantitative data showing renal expression of active β-catenin, P16^INK4A^, P19^ARF^, α-SMA and fibronectin in different groups. Numbers (1–3) indicate each individual animal in each given group. **p < 0.01, ***p < 0.001 versus 18-month-old mice group. ^##^ *p* < 0.01, ^###^ *p* < 0.001versus 21-month-old mice group alone (*n* = 5). **(L)** Representative micrographs showing the expressions of active β-catenin (top), β-galactosidase activity (SA-β-gal) (middle) and fibronectin (bottom) in kidneys in different groups. Cryosections were subjected to fluorescence staining for active β-catenin or stained for SA-β-gal. Paraffin-embedded kidney sections were immunostained with antibody against fibronectin. Arrows indicate positive staining. Scale bar, 50 μm.

To identify the effects of AMPK signaling on kidney aging, we injected the 18-month-old mice which had undergone unilateral nephrectomy with O304 for 12 weeks. The details of experimental design were showed in Figure 3A. We first assessed the expression of p-AMPK in different groups. As shown in Figure 3B, compared with 18-month-old mice, the expression of p-AMPK detected by immunohistochemical staining was down-regulated in 21-month-old mice and obviously restored by treatment of O304. Similar result was demonstrated by analysis of western blot for p-AMPK and AMPK (Figures 3C–E).

The effects of O304 on β-catenin, renal senescence and fibrosis were then assessed in the naturally aging mice model. As shown in Figures 3F–K, western blot analyses showed that the expressions of active β-catenin, P16^INK4A^, P19^ARF^, α-SMA and fibronectin were greatly increased in 21-month-old mice, but inhibited by O304 treatment. Immunofluorescent staining of active β-catenin and immunohistochemical staining of fibronectin also showed similar results (Figure 3L). Besides, the senescence-associated β-galactosidase (SA-β-gal) staining also showed that O304 treatment reversed the β-galactosidase activity which was highly expressed in 21-month-old mice (Figure 3L). These results suggested that AMPK activator could inhibit β-catenin and alleviate renal fibrosis and senescence.

### Transcriptomic Analysis Further Proves the Beneficial Effects of O304 on Autophagy, Fibrosis, Senescence and Energy Metabolism in Naturally Aging Mice

**FIGURE 4:**
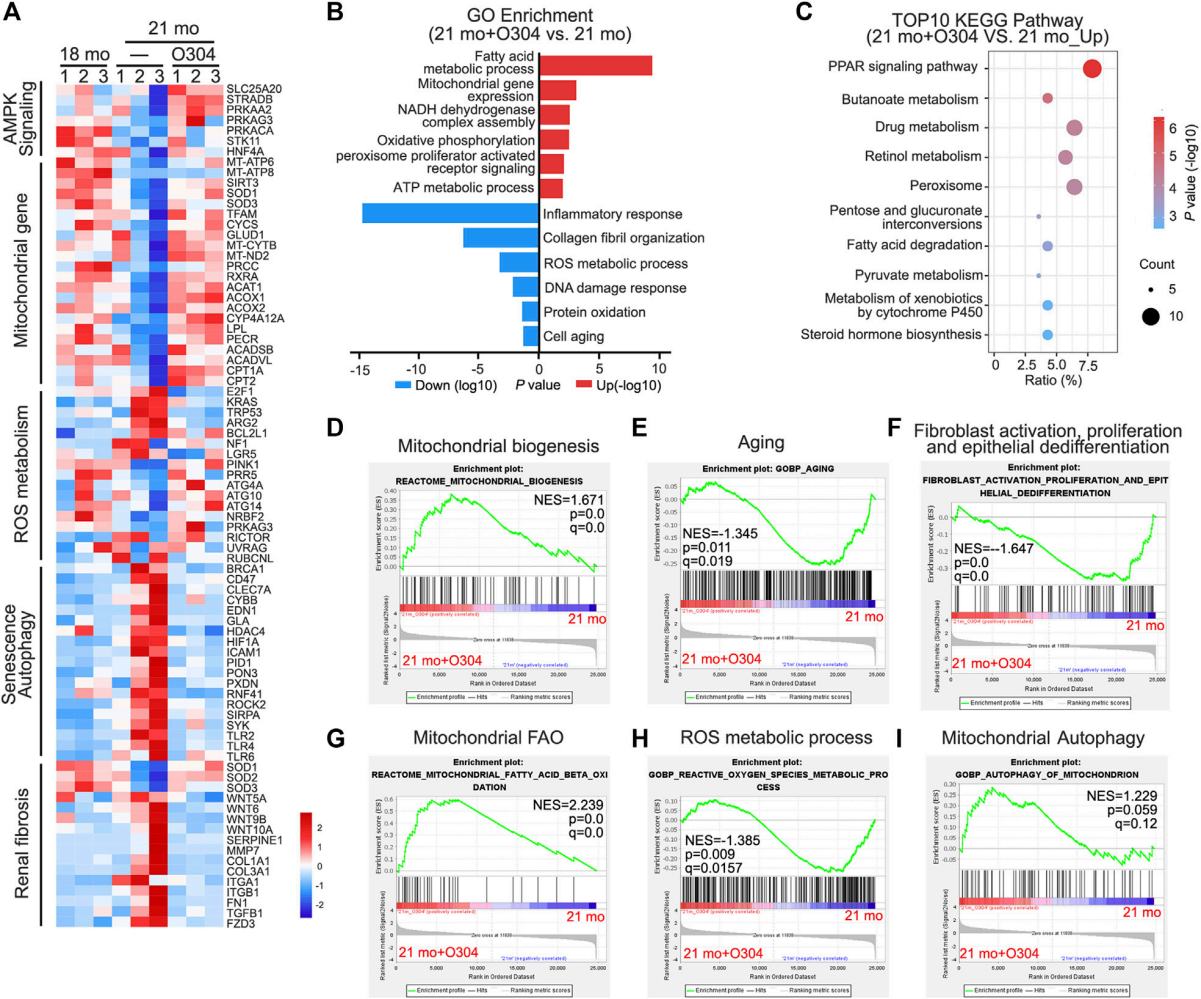
Transcriptomic analysis further proves the effects of O304 on autophagy, fibrosis, senescence and energy metabolism in naturally aging mice. **(A)** Heatmap plot of transcriptomic analysis shows the changes in the AMPK signaling, mitochondrial genes, ROS metabolism, senescence, autophagy and renal fibrosis in different groups. **(B)** Gene ontology (GO) analysis shows differentially expressed genes (DEGs) in 21mo vs 21mo/O304 mice. Red color indicates the upregulation of genes after O304 treatment, while blue color indicates the downregulation. **(C)** KEGG pathway analysis shows the top 10 target-pathways of O304 in the upregulated genes by GO analysis. **(D–I)** Gene set enrichment analysis (GSEA) showing the different enriched genes in 21-month-old group or O304 treatment group. *q* value meaning adjusted *p* value. NES, normalized enrichment score.

Next, we performed transcriptomic analysis to further exam the beneficial effects of O304 in naturally aging mice. As shown in Figure 4A, heatmap plot of transcriptomic analysis showed that 21-month-old mice were associated with the downregulation of AMPK signaling, mitochondrial function and autophagy. Besides, they were also associated with the upregulation of ROS production, renal fibrosis and senescence. However, O304 treatment significantly reversed all these changes. Then Gene ontology (GO) analysis was performed to explore the differentially expressed genes between the 21-month-old mice group (21 mo) and the O304 treatment group (21 mo/O304). As shown in Figure 4B, O304 treatment could upregulate the genes targeting fatty acid metabolism, oxidative phosphorylation and ATP metabolism, and downregulate the genes targeting inflammatory response, ROS metabolic process and cellular senescence. Since the regulation of O304 on genes mainly concentrated in mitochondrial function or energy homeostasis, KEGG pathway enrichment analysis was performed to further analyze the effect of O304 on metabolism. We found that the top 10 enriched signaling pathways included PPAR signaling pathway, butanoate metabolism, drug metabolism, retinol metabolism, peroxisome, pentose and glucuronate interconversions, fatty acid degradation, pyruvate metabolism, metabolism of xenobiotics by cytochrome P450 and steroid hormone biosynthesis (Figure 4C), among which PPAR signaling pathway was the topmost one. Finally, Gene set enrichment analysis (GSEA) further demonstrated that O304 treatment could inhibit renal senescence, renal fibrosis and ROS production, and activate mitochondrial biogenesis, mitochondrial fatty acid oxidation (FAO) and autophagy (Figures 4D–I).

### O304 Could Promote Fatty Acid Metabolism in Naturally Aging Mice

**FIGURE 5:**
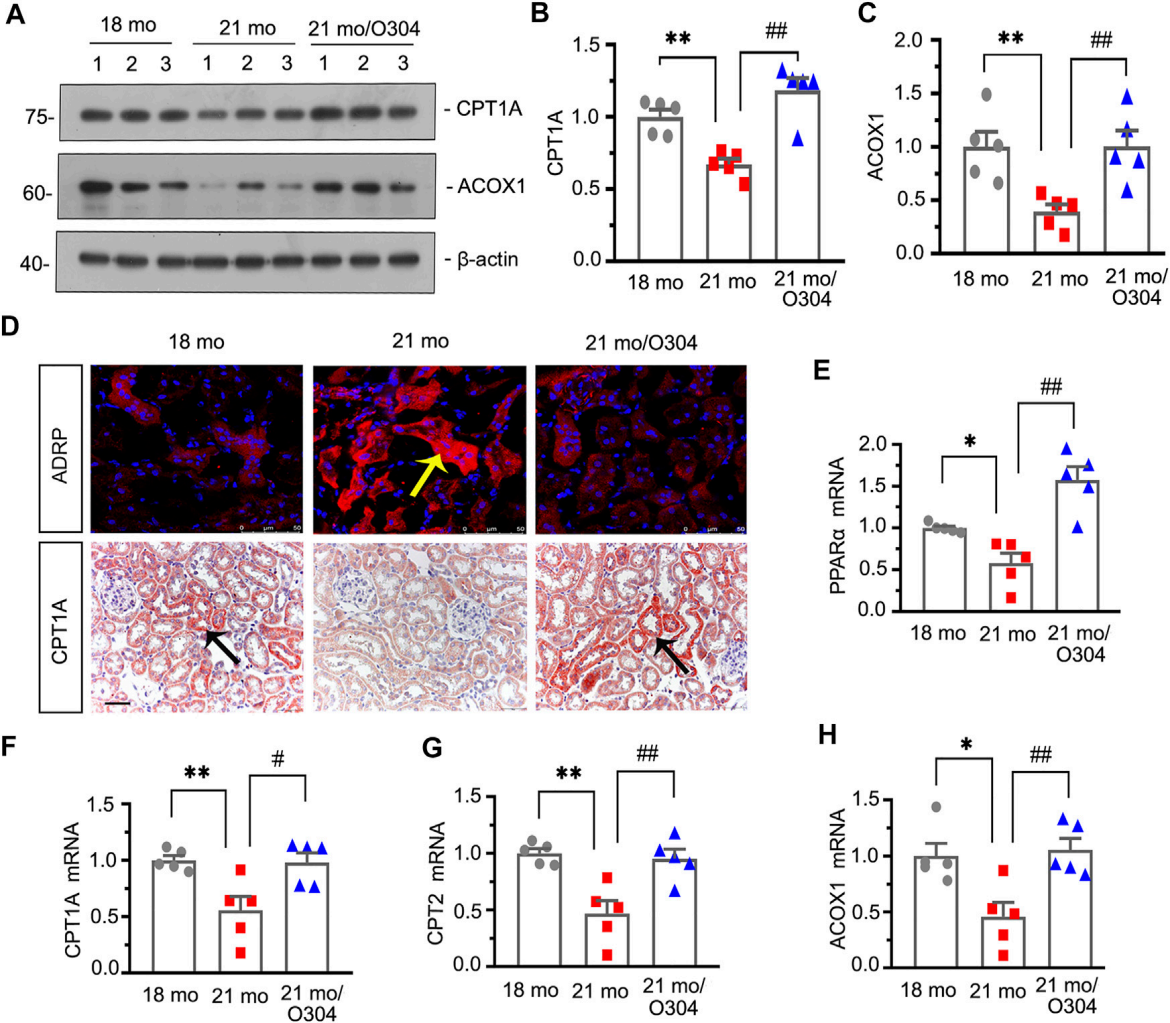
O304 could promote fatty acid metabolism in naturally aging mice. **(A–C)** Representative western blot and quantitative data showing renal expression of CTP1A and ACOX1 in different groups. Numbers (1–3) indicate each individual animal in each given group. **p < 0.01 versus 18-month-old mice group. ^##^ *p* < 0.01 versus 21-month-old mice group alone (*n* = 5). **(D)** Representative micrographs showing ADRP (top) and CPT1A (bottom) expression in kidneys in different groups. Cryosections were subjected to fluorescence staining for ADRP. Paraffin-embedded kidney sections were immunostained with an antibody against CPT1A. Arrows indicate positive staining. scale bar, 50 μm. **(E–H)** Graphical representations show the relative abundance of PPARα, CPT1A, CPT2 and ACOX1 mRNA in different groups. *p < 0.05, **p < 0.01 versus 18-month-old mice group. ^#^ *p* < 0.05, ^##^ *p* < 0.01versus 21-month-old mice group alone (*n* = 5).

Transcriptomic analysis demonstrated the regulatory effect of O304 on energy metabolism, in which PPAR signaling pathway was involved. Then we further assessed the effects of O304 on the fatty acid metabolism. As shown in Figures 5A–C, western blot analyses showed that the expressions of CPT1A and ACOX1 were decreased in 21-month-old mice, but restored after O304 treatment. Immunohistochemical staining of CPT1A also showed similar results (Figure 5D). Besides, immunofluorescent staining showed that the upregulation of ADRP in 21-month-old mice was inhibited by O304 treatment (Figure 5D). What’s more, the expressional levels of peroxisome proliferator–activated receptor-α (PPARα), CPT1A, carnitine palmitoyl transferase-2 (CPT2) and ACOX1 were assessed by quantitative real-time PCR and the results showed that the downregulation of their expressions in 21-month-old mice was restored after O304 treatment (Figures 5E–H). All these results confirmed that O304 could promote fatty acid metabolism.

### O304 Could Promote Autophagy and Alleviate Mitochondrial Dysfunction in Naturally Aging Mice

**FIGURE 6:**
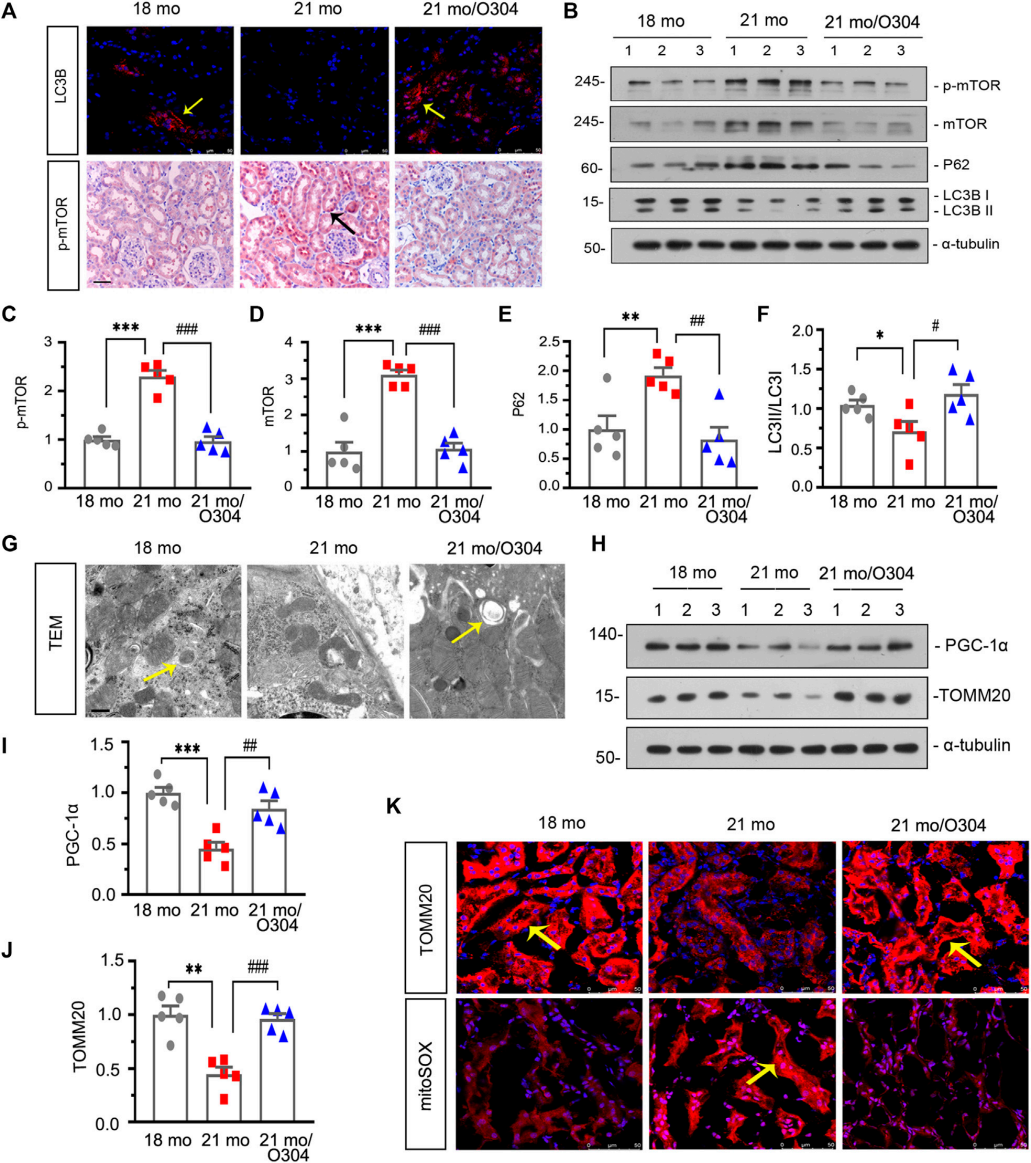
O304 could promote autophagy and alleviate mitochondrial dysfunction in naturally aging mice. **(A)** Representative micrographs showing LC3B (top) and p-mTOR (bottom) expression in kidneys in different groups. Cryosections were subjected to fluorescence staining for LC3B. Paraffin-embedded kidney sections were immunostained with an antibody against p-mTOR. Arrows indicate positive staining. scale bar, 50 μm. **(B–F)** Representative western blot and quantitative data showing renal expression of p-mTOR, mTOR, P62 and enhanced LC3-II formation in different groups. Numbers (1–3) indicate each individual animal in each given group. *p < 0.05, **p < 0.01, ***p < 0.001 versus 18-month-old mice group. ^#^ *p* < 0.05, ^##^ *p* < 0.01, ^###^ *p* < 0.001 versus 21-month-old mice group alone (*n* = 5). **(G)** Representative transmission electron microscopy (TEM) micrographs show autophagic vacuole in different groups in ultrathin kidney sections. Arrow indicates autophagic vacuole. Scale bar, 0.5 μm. **(H–J)** Representative western blot and quantitative data showing renal expression of PGC-1α and TOMM20 in different groups. Numbers (1–3) indicate each individual animal in each given group. **p < 0.01, ***p < 0.001 versus 18-month-old mice group. ^##^ *p* < 0.01, ^###^ *p* < 0.001versus 21-month-old mice group alone (*n* = 5). **(K)** Representative micrographs showing the expression of TOMM20 (top) and mitochondrial ROS by mitoSOX staining (bottom) in kidneys in different groups. Cryosections were subjected to fluorescence staining for TOMM20 and mitoSOX. Arrows indicate positive staining.

We next assessed the effect of O304 on autophagy. As shown in Figure 6A, compared with 18-month-old mice, the expression of LC3B detected by immunofluorescent staining was down-regulated in 21-month-old mice, however, O304 treatment reversed its expression. On the other hand, immunohistochemical staining showed that the upregulation of p-mTOR in 21-month-old mice was inhibited by O304 treatment. Besides, western blot analyses showed that the expressional levels of p-mTOR, mTOR and P62 (Figures 6B–E) were greatly increased in 21-month-old mice, while the conversion of LC3-I to LC3-II (Figures 6B,F) was down-regulated, compared with 18-month-old mice. However, O304 treatment reversed all of their expressions (Figures 6B–E). In addition, compared with 21-month-old mice, autophagic vacuole could obviously be detected by transmission electron microscopy in 18-month-old mice and O304 treated mice (Figure 6G).

We then examined the effects of O304 on mitochondrial dysfunction. As shown in Figures 6H–J, western blot analyses showed that the expressional levels of peroxisome proliferator-activated receptor-γ coactivator-1α (PGC-1α) and TOMM20 were decreased in 21-month-old mice, but were up-regulated again after O304 treatment. Similar result was demonstrated by immunohistochemical staining to assess the expression of TOMM20 (Figure 6K). On the other hand, the mitoSOX probe staining showed that mitochondrial ROS production was increased in 21-month-old mice and greatly inhibited by O304 treatment (Figure 6K). These results demonstrated that O304 could alleviate mitochondrial dysfunction.

We also examined the effects of O304 on kidney function. The expressional levels of serum creatinine and serum urea were examined, but there was no statistical difference among different groups (Supplementary Figures S1A,B).

### O304 Could Activate AMPK and Promote Fatty Acid Metabolism *In vitro*

**FIGURE 7:**
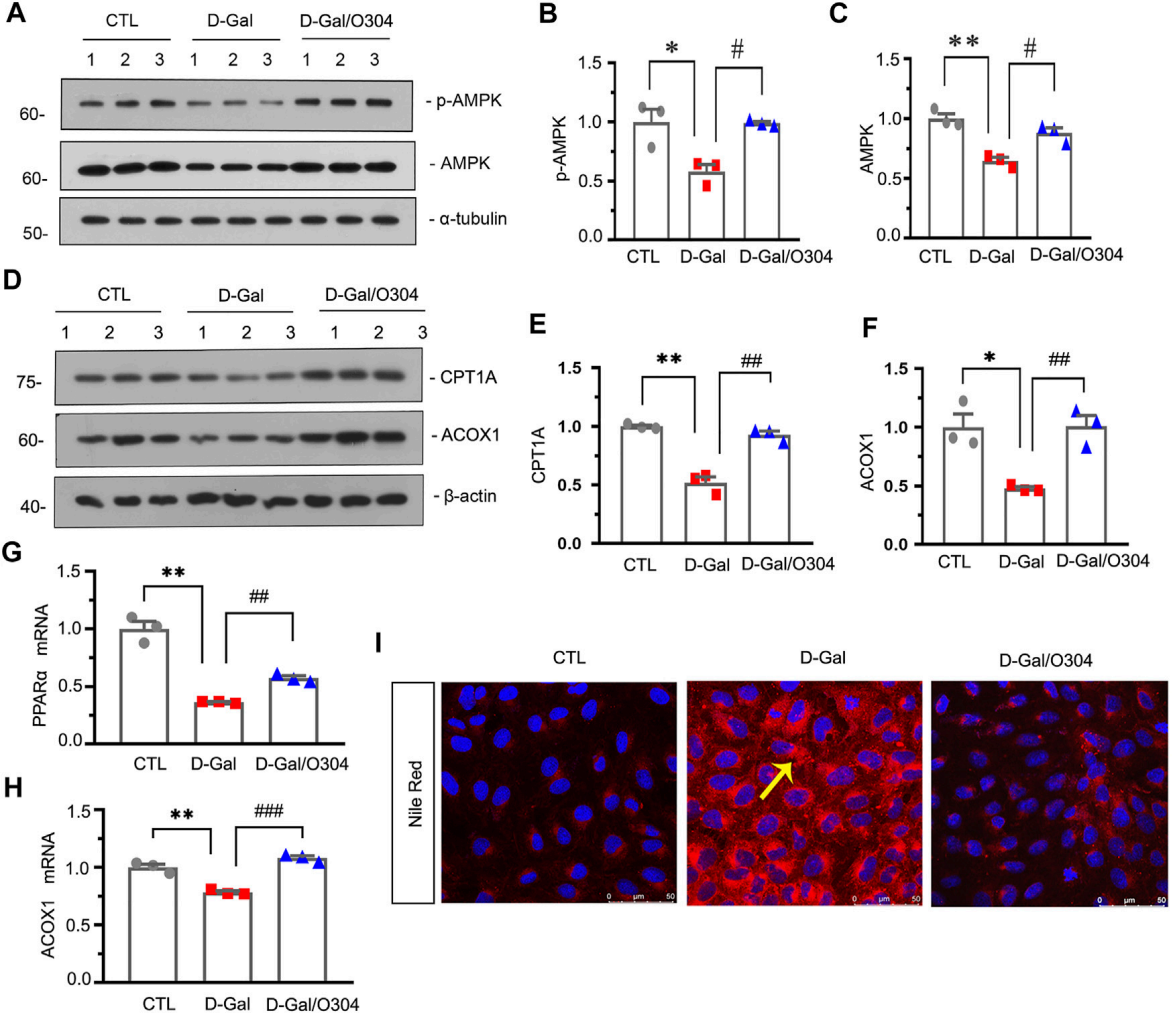
O304 could activate AMPK and promote fatty acid metabolism *in vitro*. **(A–C)** Representative western blot showing the expression of p-AMPK and AMPK in different groups. HKC-8 cells were stimulated by D-Gal (10 mg/ml) for 60 h with or without O304 (50 nM). *p < 0.05, **p < 0.01 versus control (Ctrl) cells; ^#^ *p* < 0.05 versus D-Gal-treated group alone (*n* = 3). **(D–F)** Representative western blot and quantitative data showing the expression of CPT1A and ACOX1. *p < 0.05, **p < 0.01 versus control group; ^##^ *p* < 0.01 versus D-Gal-treated group alone (*n* = 3). **(G,H)** Graphical representations show the relative abundance of PPARα and ACOX1 mRNA in different groups. **p < 0.01 versus control group; ^##^ *p* < 0.01, ^###^ *p* < 0.001 versus D-Gal-treated group alone (*n* = 3). **(I)** Representative micrographs showing the formation of fat in HKC-8 cells by Nile red staining in different groups. Arrows indicate positive staining of fatty formation.

We then investigated the effects of O304 on AMPK and fatty acid metabolism *in vitro*. Firstly, HKC-8 cells, a renal proximal tubular cell line, were pretreated with O304, followed by administration of D-gal. Western blot analyses showed that the expressional levels of p-AMPK, AMPK, CPT1A and ACOX1 were significantly inhibited by D-gal, but restored after O304 treatment (Figures 7A–F). Besides, the results of quantitative real-time PCR showed that O304 reversed the downregulation of PPARα and ACOX1 induced by D-Gal (Figures 7G,H). In addition, Nile red staining also showed that the accumulation of fat induced by D-Gal was also inhibited by O304 treatment. (Figure 7I). All these results confirmed that O304 could promote fatty acid metabolism *in vitro*.

### O304 Could inhibit β-catenin and Alleviate Renal Senescence and Fibrosis *In vitro*

**FIGURE 8:**
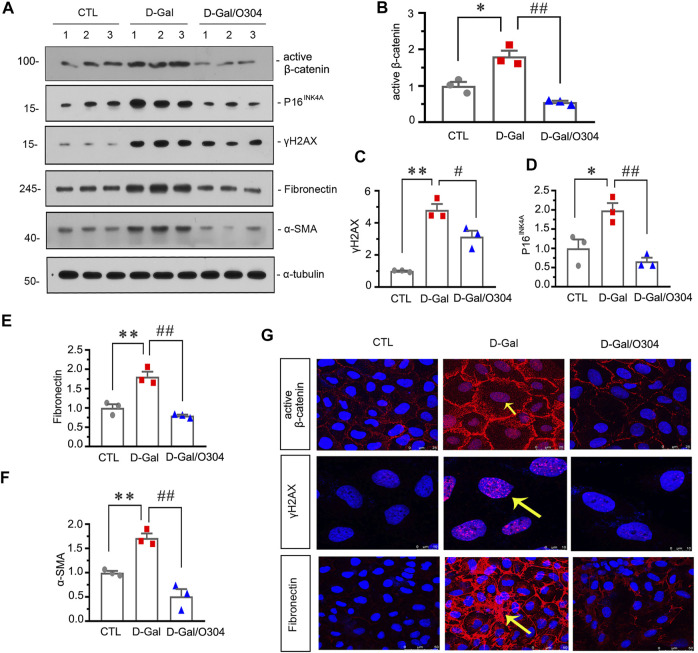
O304 could inhibit β catenin and alleviate renal senescence and fibrosis *in vitro*. **(A–F)** Representative western blot and quantitative data showing the expression of active β-catenin, P16^INK4A^, γH_2_AX, fibronectin and α-SMA in HKC-8 cells. HKC-8 cells were pretreated with O304 at 50 nM for 1 h, followed by treatment with D-Gal at 10 mg/ml for 60 h *p < 0.05, **p < 0.01 versus the control group alone; ^#^ *p* < 0.05, ^##^ *p* < 0.01 versus the D-Gal-treated group alone (*n* = 3). **(G)** Representative micrographs showing the expression of active β-catenin (top), γH_2_AX (middle) and fibronectin (bottom) in HKC-8 cells. HKC-8 cells cultured on coverslips were immunostained with an antibody against active β-catenin, γH2AX or fibronectin. Arrows indicate positive staining.

Next, the effects of O304 on β-catenin and renal senescence and fibrosis were also assessed *in vitro*. As shown in Figures 8A–F, administration of D-gal obviously up-regulated the expression of active β-catenin, P16^INK4A^, γH2AX, fibronectin and α-SMA, but O304 treatment significantly inhibited all of their abnormal expression. Similar results were demonstrated when the expressional levels of active β-catenin, fibronectin and γH2AX were analyzed by immunofluorescence staining (Figure 8G). These results suggested that O304 could inhibit β-catenin and alleviate cellular fibrosis and senescence *in vitro*.

### O304 Could Promote Autophagy and Alleviate Mitochondrial Dysfunction *In vitro*

**FIGURE 9:**
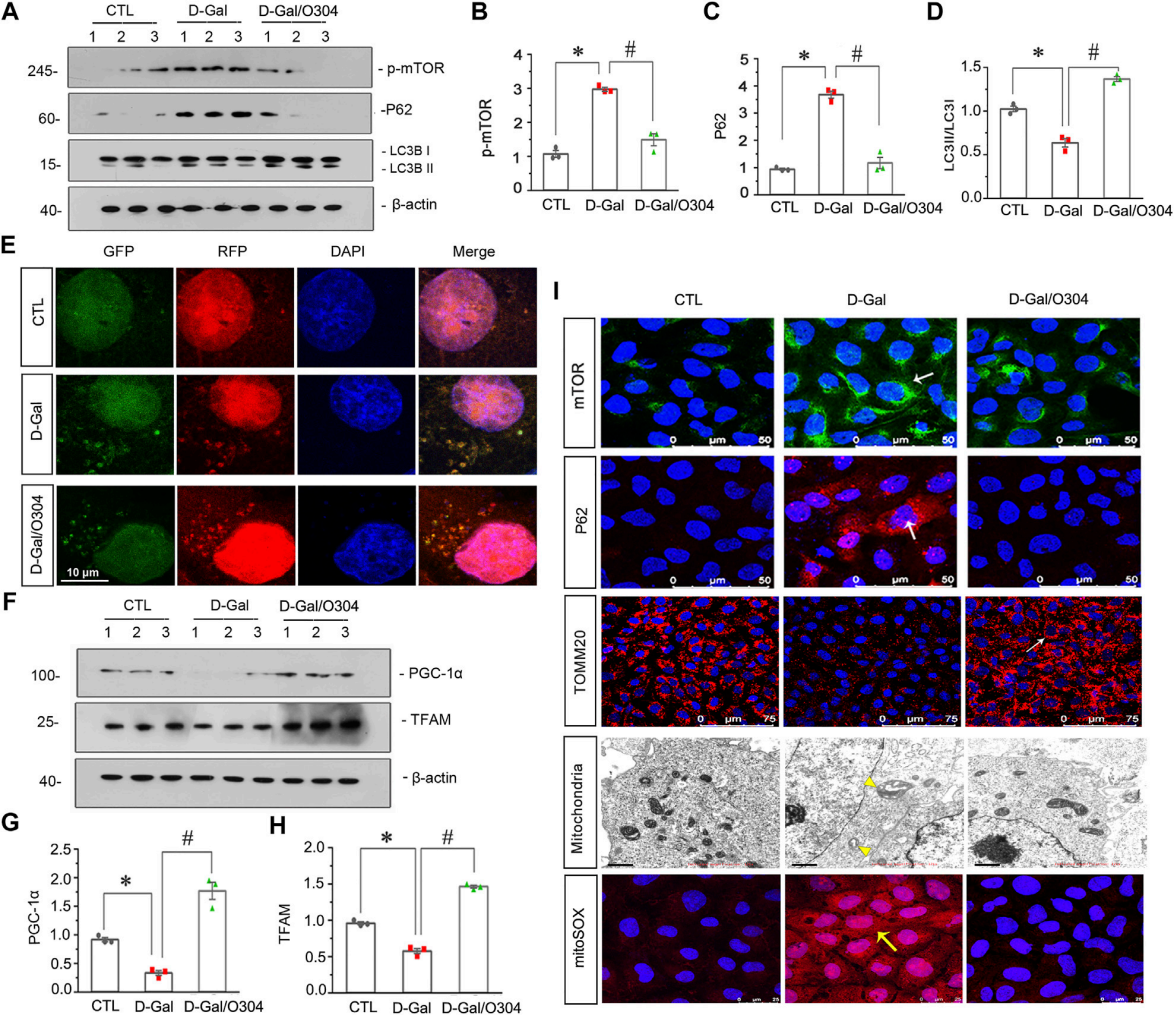
O304 could promote autophagy and alleviate mitochondrial dysfunction *in vitro*. **(A–D)** Representative western blot showing the expression of p-mTOR, P62, and LC3B in different groups. HKC-8 cells were stimulated by D-Gal (10 mg/ml) for 60 h with or without O304 (50 nM). *p < 0.05 versus control (Ctrl) cells; ^#^ *p* < 0.05 versus D-Gal-treated group alone (*n* = 3). **(E)** Representative micrographs show that O304 promoted acidic-pH LC3B-positive autophagolysosomes (red fluorescence). HKC-8 cells were pre-transfected with lentiviruses expressing RFP-GFP-LC3B for 12 h, and were then stimulated by D-Gal (10 mg/ml) for 60 h with or without O304 (50 nM). Natural-pH LC3B-positive autophagosomes (green fluorescence) and acidic-pH LC3B-positive autophagolysosomes (red fluorescence) were detected. **(F–H)** Representative western blot and quantitative data showing the expression of PGC-1α and TFAM. *p < 0.05 versus control group; ^#^ *p* < 0.05 versus D-Gal-treated group alone (*n* = 3). **(I)** Representative micrographs showing the expression of mTOR, P62, TOMM20 and mitochondrial ROS production in different groups. HKC-8 cells were seeded on coverslips and stimulated by D-Gal (10 mg/ml) for 60 h with or without O304 (50 nM). The cells were immunostained with mitoSOX probe or antibodies against mTOR, P62 and TOMM20, respectively. Representative transmission electron microscopy (TEM) micrographs (middle) showing O304 protects the normal structure of mitochondria in HKC-8 cells. For mTOR, P62, TOMM20 and mitoSOX staining, arrows indicate positive staining. For TEM analyses, arrowheads indicate abnormal-shaped mitochondria.

Then the effects of O304 on autophagy and mitochondrial dysfunction were assessed *in vitro*. As shown in Figures 9A–C, D-Gal stimulation promoted the upregulation of p-mTOR and the autophagosome-selective substrate, P62. However, their expressions were obviously inhibited by co-treatment with O304. Similar results were observed when the expressional levels of mTOR and P62 was assessed by immunofluorescent staining (Figure 9I). Besides, the conversion of LC3B-I to LC3B-II was also greatly increased by co-treatment with O304 in D-Gal-treated cells (Figures 9A,D). To further assess autophagic flux, HKC-8 cells were infected with lentiviruses expressing a tandem RFP-GFP-LC3B fusion protein, and were then treated with D-Gal or were co-treated with D-Gal/O304. The RFP-GFP-LC3B sensor enables LC3B-positive, neutral-pH autophagosomes as green fluorescence, whereas LC3B-positive acidic pH autophagolysosomes exhibit red fluorescence. As shown in Figure 9E, D-Gal induced autophagosome accumulation without fusion of autophagsomes to lysosomes, as indicated by their golden color (overlap of red and green fluorescence). However, co-treatment with O304 triggered autophagic flux in HKC-8 cells, as shown by red puncta due to autophagosomes colocalizing with lysosomes.

We also examined the effects of O304 on mitochondrial dysfunction. As shown in Figures 9F–H, O304 significantly restored the down-regulation of the PGC-1α and mitochondrial transcription factor A (TFAM) induced by D-Gal, which were two key factors of mitochondrial biogenesis. Similar results were observed when TOMM20 was assessed by immunofluorescent staining, suggesting the protective effects of O304 on mitochondrial mass (Figure 9I). Consistently, the ultrastructure of mitochondria which was assessed by transmission electron microscopy (TEM) further clarified the protective effects of O304. As shown in Figure 9I, D-Gal induced swollen mitochondria with disorganized and fragmented cristae in HKC-8 cells, however, co-treatment with O304 greatly improved the normal structure of mitochondria. Besides, mitochondrial ROS production detected by mitoSOX probe staining also showed that the upregulation of ROS production induced by D-Gal was also greatly inhibited after O304 treatment (Figure 9I). These results suggested that O304 could promote autophagy and alleviate mitochondrial dysfunction *in vitro*.

### O304 Promotes Autophagic Flux *In vitro*

**FIGURE 10:**
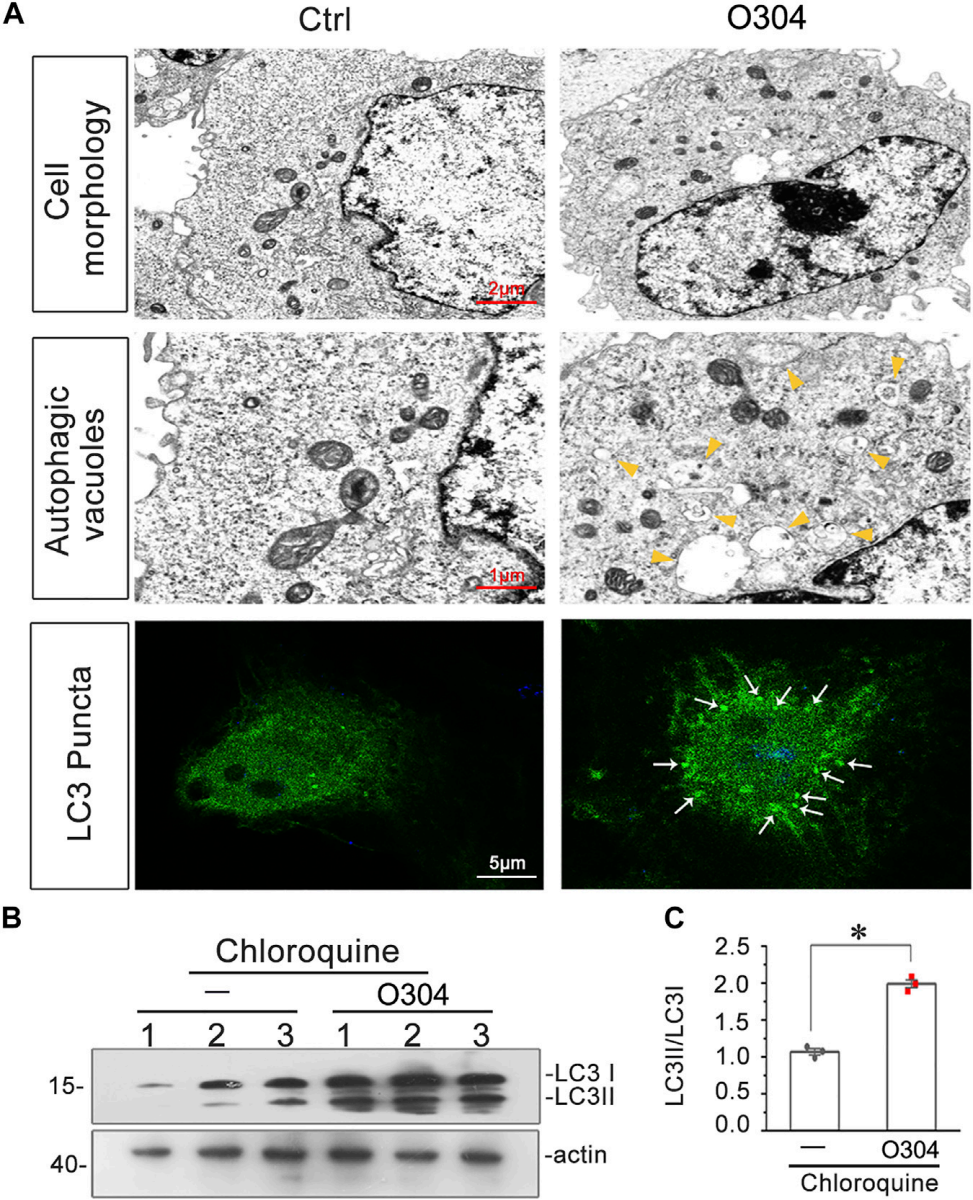
O304 promotes autophagic flux *in vitro*. **(A)** Representative electron microscopy (TEM) micrographs show that O304 increased autophagic vacuoles (yellow arrowheads). HKC-8 cells were treated with O304 (50 nM) for 60 h. Representative micrographs showO304 enhanced LC3B formation. HKC-8 cells were transiently transfected with GFP-LC3B plasmid for 24 h, and were then stimulated with or without O304 (50 nM) for 60 h. The cells were observed by fluorescence microscopy. **(B,C)** Representative western blot shows that O304 enhanced LC3BII formation. HKC-8 cells were treated with chloroquine (50 μM) and co-treated with or without O304 (50 nM) for 60 h *p < 0.05 versus chloroquine alone (*n* = 3).

Finally, the effects of O304 on autophagic flux were detected. As shown in Figure 10A, TEM images demonstrated that, compared with normal cultured cells, O304 incubation induced an increase in autophagic vacuoles in HKC-8 cells. Furthermore, immunostaining for LC3B exhibited the formation of more LC3B puncta in O304-treated group. We next assessed the effects of chloroquine on O304-treated cells. As shown in Figures 10B,C, co-treatment of O304 with chloroquine significantly enhanced the formation of LC3B, which suggested an increase in autophagic flux by O304.

## Discussion

Aging are becoming great challenges for public health since aging population is growing rapidly worldwide (Beard and Bloom, 2015). Elderly people (over 60 years) will account for 11–22% of the population by 2050 according to data from the World Health Organization (WHO) (Nabavi et al., 2016). Kidney is one of the most important organs in the body, and aging plays an important role in the high morbidity of kidney injury and declined kidney function (Inker et al., 2015; Minutolo et al., 2015; Fang et al., 2020). Among the etiology of aging, energy metabolism imbalance is highly related with the loss of function in all organs, especially kidney (Miao et al., 2019).

The kidney is one of the most energy-demanding organs in order to remove waste from the blood and regulate fluid and electrolyte balance (Bhargava and Schnellmann, 2017). Research showed that the deficiency of energy supply highly contributes to the decline of kidney function (Emma et al., 2016). Notably, mitochondria are responsible for energy production. Hence, mitochondrial homeostasis plays a key role in maintaining normal function of kidney (Jiang et al., 2020).

Autophagy is a self-degradative process and plays important roles in energy supply and aging (Glick et al., 2010). It is necessary for organellar quality control by degrading damaged or misfolded proteins and even damaged mitochondria, the latter of which is also called mitophagy (Chen et al., 2020). When the energy supply is deficient, autophagy is a critical process in maintaining energy homeostasis as both extra- and intra-cellular components can be further used to produce ATP through catabolic reactions (Yang et al., 2019). Intact autophagic machinery promotes longevity and alleviates age-related phenotypes, while reduced autophagic activity is associated with aging acceleration (Rubinsztein et al., 2011).

AMPK is a central energy metabolic regulator. In case of energy deficiency, AMPK is activated and targets a range of physiological processes, which will cause the increase in energy production and a coordinated decrease in ATP usage through mitochondrial biogenesis and increased metabolism. In addition to energy supply, AMPK also highly involves in autophagy through inhibiting mTOR (Kim et al., 2011; Li and Chen, 2019). However, the role of AMPK activation in kidney aging has not been investigated. O304 is a direct pan-AMPK activator which was identified the beneficial role in glucose homeostasis type 2 diabetes patients (Steneberg et al., 2018). However, its therapeutic potential in kidney aging has not been clarified.

In this study, we found that O304 beneficially protects against kidney aging. There are several lines to support our findings. First, we tested the role of O304 in naturally aging mice. Results show that O304 effectively inhibited cellular senescence and age-related kidney fibrosis (Figure 3). Furthermore, O304 restored fatty acid metabolism, promoted autophagy and maintained mitochondrial homeostasis (Figures 5, 6). These results were also proved by transcriptomic sequencing analysis (Figure 4). The therapeutic effects of O304 were also proved in D-Gal-induced tubular cell culture (Figures 7–9). Interestingly, we also found that O304 solely triggered autophagic activity in renal tubular cells (Figure 10), further suggesting its potent potential for therapeutic effects on kidney aging. We also found that Wnt/β-catenin signaling could be inhibited by O304. Wnt/β-catenin signaling is an evolutionarily conserved pathway involved in organ development and tissue repair, and it often keeps silent in normal adults, but would be reactivated after kidney injury in a wide range of CKD models (Luo et al., 2018). It has been proved that Wnt/β-catenin signaling is closely related to mitochondrial dysfunction and kidney aging (Miao et al., 2019) and fatty acid metabolism (Behari et al., 2014; Feng et al., 2019). In this study, we demonstrated that O304 is able to inhibit Wnt/β-catenin signaling, revealing that O304 may act through multiple pathways.

In summary, we show that AMPK activator O304 can alleviate kidney aging through inducing energy metabolism, promoting autophagy and maintaining mitochondrial homeostasis. Although more studies are needed, our study suggests that O304 may represent a novel therapeutic potential to renal tubular senescence and age-related kidney injury.

## Data Availability

The datasets presented in this study can be found in online repositories. The names of the repository/repositories and accession number(s) can be found below: Gene Expression Omnibus, accession number GSE192512.

## Supplementary Material

Supplementary Figure S1 (A,B) Graphical representation of the levels of serum creatinine and serum urea in different groups. ns, none of significance (*n*=5).
